# Improving Post-Filtering of Artificial Speech Using Pre-Trained LSTM Neural Networks

**DOI:** 10.3390/biomimetics4020039

**Published:** 2019-05-28

**Authors:** Marvin Coto-Jiménez

**Affiliations:** Escuela de Ingeniería Eléctrica, Universidad de Costa Rica, San José 11501-2060, Costa Rica; marvin.coto@ucr.ac.cr

**Keywords:** deep learning, LSTM, machine learning, post-filtering, signal processing, speech synthesis

## Abstract

Several researchers have contemplated deep learning-based post-filters to increase the quality of statistical parametric speech synthesis, which perform a mapping of the synthetic speech to the natural speech, considering the different parameters separately and trying to reduce the gap between them. The Long Short-term Memory (LSTM) Neural Networks have been applied successfully in this purpose, but there are still many aspects to improve in the results and in the process itself. In this paper, we introduce a new pre-training approach for the LSTM, with the objective of enhancing the quality of the synthesized speech, particularly in the spectrum, in a more efficient manner. Our approach begins with an auto-associative training of one LSTM network, which is used as an initialization for the post-filters. We show the advantages of this initialization for the enhancing of the Mel-Frequency Cepstral parameters of synthetic speech. Results show that the initialization succeeds in achieving better results in enhancing the statistical parametric speech spectrum in most cases when compared to the common random initialization approach of the networks.

## 1. Introduction

Text-to-speech synthesis (TTS) is the technique of generating intelligible speech from a specific text. Applications of TTS have evolved over time, as the quality of the systems has improved to encompass virtual assistants, in-car navigation systems, e-book readers and communicative robots [1]. In the present and future applications, any task that requires the transfer of information between people and machines, or between people with a device as a communication intermediary, becomes a potential area of pertinence [2].

In recent years, TTS systems have progressed from the capacity of producing intelligible speech to the more difficult challenge of generating voices with natural sound, in multiple languages. Despite these trends, there are unresolved obstacles, such as improving the overall quality in the voices generated with concatenated segments of speech and in the past two decades with statistical parametric methods.

The statistical methods have grown in popularity since they arose in the late 1990s [3], particularly those based on Hidden Markov Models (HMMs). HMMs are known for their flexibility in changing speaker characteristics, having a low footprint, and for their capacity to produce advanced features such as average voices [4] and accent modification [5]. Nowadays they are still a preferred technique for several languages and conditions [6].

The main shortcoming of the HMM-based speech synthesis is its quality. It is well known that the generated sequences of parameters from the HMMs are temporally smoothed, producing perceptual differences between synthetic and natural speech. There have been several attempts to improve the quality of synthesized speech, based on Deep Learning approaches: The first main approach is to substitute the HMM for deep neural networks (DNN) [7,8,9,10], learning the map between linguistic specification directly to speech parameters. The second approach is to apply post-filters for the parameters generated by the HMMs [11,12,13]. The post-filters are usually implemented with DNN, modeling the conditional probability of the acoustic differences between artificial and natural speech.

The main reason to apply DNN is the benefits obtained in many related areas, where the enhancing of signals represents important challenges. For example, to provide better speech recognition in adverse conditions or environments [14,15], and the implementation in low power consumption systems [16].

The high computational cost of training is a significant difficulty in applying some kinds of DNN. For some types of networks, such as Long-Short-term Memory Networks (LSTM), the computational cost has been a shortcoming for the experimentation with more hidden layers or units in the networks [17,18]. With the aim of searching for more efficient ways of training the networks, recent experiences have tested variants of well-known models [19], or the use of extreme learning to explore new conditions [20].

In this work, we explore the benefits of supervised pre-training of LSTM networks as post-filters for spectrum data of synthetic voices generated with HMM, in comparison with the usual random weight initialization. This initialization is performed in the form of an auto-associative network.

### 1.1. Related Work

Recent experimental results with DNN architectures have been obtained with initialization or training schemes different from the classical feedforward neural networks [21]. For example, unsupervised pre-training that initializes the parameters in a better basin of attraction of the optimization procedure.

In the field of speech technologies, Restricted Boltzmann Machines have been unsupervised initialized and then fine-tuned for speech recognition [22]. The breakthrough to effective training strategies for deep networks came with the algorithms for training deep belief networks, based on a greedy layer-wise unsupervised pre-training followed by supervised fine-tuning [23]. Benefits of the unsupervised pre-training have also been verified in other fields, such as music classification [24] and visual recognition [25]. Semi-supervised techniques applied in similar applications [26] combine at least one stage of unlabeled data to initialize the neural networks.

In artificial speech enhancement, several proposals of neural networks implemented as post-filters have been presented [27], to ensure that the speech parameters of the artificial voices become similar to those of natural speech. A similar approach was presented in [28,29], and enhancement of spectral parameters has been implemented in [30].

In [31], the spectrum features of the synthesized speech are enhanced using networks such as DBNs, and also RBM has been previously studied [29]. More recently, the use of Recurrent Neural Networks (RNNs) was presented in [32], in contrast to standard feedforward networks or models like Deep Belief Nets for post-filtering synthesized speech. The inherent structure of RNNs seems to deal better with the time-dependent nature of the speech signal, which has also been noted in [28]. Previous work using recurrent LSTM networks for the enhancement of the Mel-cepstral coefficients of synthetic voices was recently presented in [18].

In these references, the most common approach is to enhance the spectral components of the synthetic speech, by mapping them to those of the original ones, using diverse deep learning algorithms. A more recent approach that considers a single-stage of multi-stream post-filters has been presented in [33,34], with greater success than the single post-filters based on LSTM.

None of the most recent proposals have made use of pre-training the networks for speech applications. Contrasting with the image classification and voice or music recognition, the post-filtering for synthesized speech is not a classification problem. Here, a regression approach is applied, which has not been previously tested with pre-training methods. In our proposal, we present a supervised initialization in an auto-associative network for the LSTM autoencoders applied to the enhancement of artificial speech. The supervised initialization, due to the data type, is more natural and provides the networks with a better start for the regression performed in the post-filtering procedure.

The benefits of initialization should also be tested in terms of the quality of synthetic speech, using common measures to assess the spectrum of the signals. None of the previous references with initialization and synthetic speech have implemented such measures. In speech enhancing and related tasks, among the time-domain measures, the most common is the Perceptual Evaluation of Speech Quality (PESQ) [35], which has been presented as an important alternative to costly and time/resource consuming subjective evaluations.

### 1.2. Problem Statement

In the analysis of natural speech, the trajectories of parameter values (e.g., MFCC, $f_{0}$, energy, aperiodic coefficients) exhibit rich modulation characteristics. Conversely, in HMM-based statistical parametric speech synthesis, the speech parameters are overly smoothed due to the statistical modeling and averaging [11,36].

For the task of enhancing the results of HMM-based speech synthesis, we may consider the speech parameters, $R_{Y}$, of synthetic utterances as a corrupted or noisy version of the parameters, $R_{X}$, of the same original utterances. In a frame-by-frame alignment of synthesized and natural speech, every frame of natural and synthetic speech is parametrized, resulting in a vector:

(1) $$c=[c_{1},c_{2},\cdots ,c_{M}]$$

where *M* is the number of extracted coefficients. $c_{m}$ can be any kind of parameters, such as $f_{0}$, energy or spectrum. For the purpose of this work, we will consider only the MFCC coefficients of both natural and synthetic speech. The analysis of every speech utterance produces a matrix of size M×T (where *T* is the number of frames extracted from any utterance) of the form

(2) $$R=[c_{1}^{⊤},c_{2}^{⊤},\cdots ,c_{T}^{⊤}]$$

With this notation, let $R_{Y}$ and $R_{X}$ be the matrices for the synthetic and natural speech respectively (both M×T size), and $R_{W}$ the concatenation of them.

In DNN-based post-filtering, we can enhance the spectral MFCC features of a synthetic voice by estimating a function $f_{\theta }$ from the data, which maps synthetic (noisy) features to natural (clean) features, with some set of weights θ of the network. This can be achieved by minimizing the function [32]:

(3) $$E\left(R_{W}\right)=\|f_{\theta }\left(R_{Y};R_{W}\right)-R_{X}{\|}^{2}$$

During the training of the DNN-based post-filter, the initial set of weights of the network $\theta_{i}$ are updated each epoch until a stop criteria. In the most traditional approach, a random set of weights became $\theta_{i}$, and each training epoch updates those weights according to the pair of artificial and natural parameters presented, to a possible set of completely different weights $\theta_{f}$ at the end of the procedure. With this set $\theta_{f}$ it is expected that spectral parameters of any synthetic utterance $R_{y}$ can be mapped through the neural network to $R_{x}$, which presents more natural characteristics.

With the initialization in the form of an auto-associative network, which learns the mapping of identity function from its inputs to its outputs, we began with a set of parameters $\theta_{A}$ that are close of those of $\theta_{f}$, due to the nature of the regression problem, where $R_{Y}$ and $R_{X}$ are not completely different and share some characteristics. In fact, both contain the same set of sounds with the same speech rate but are a pair of natural and synthetic speech.

We pretend to show that $\theta_{A}$ is a better initialization for the LSTM post-filters than $\theta_{R}$. To show this fact, we propose several experiments with the aim of answering the following questions: (I) Do the benefits of *a* depend on what data has been used for initialization? (II) Can these benefits be detected with measures of signal quality, and not only with measures of the training procedure itself?

To our knowledge, this is a novel way to initialize, employ and evaluate the LSTM post-filters for synthetic speech. The rest of this chapter is organized as follows: Section 2 provides some details of the LSTM neural networks, of importance in the modeling of MFCC post-filtering. Section 3 describes with detail the Materials and Methods that are part of the proposal. Section 4 presents the Results, whilst Section 5 shows the Discussion of the results. Finally, conclusions are given in Section 6.

## 2. Long Short-Term Memory Recurrent Neural Networks

Among the many new algorithms developed to improve some tasks related to speech, such as speech synthesis recognition, several groups of researchers have experimented with the use of DNN, with encouraging results. Deep learning, based on several kinds of neural networks with many hidden layers, have achieved important results in many machine learning and pattern recognition problems. The disadvantage of using such networks is that they cannot directly model the dependent nature of sequential parameters, something which is desirable to imitate human speech production. It has been suggested that one way to solve this problem is to include RNN [37,38] in which there is feedback from some of the neurons in the network, either backward or to themselves, forming a kind of memory that retains information about previous states.

An extended kind of RNN, which can store information over long or short time intervals, has been presented in [39] and is called LSTM. Recently, LSTM was successfully used in speech recognition as well as in other applications to speech recognition [17,40,41] and classification [42,43]. The storage and use of long-term and short-term information within the network are potentially significant for many applications, including speech processing, non-Markovian control, and music composition [39].

In an LSTM network with several layers of units with memory, output vector sequences $y=\left(y_{1},y_{2},\cdots ,y_{T}\right)$ are computed from input vector sequences $x=\left(x_{1},x_{2},\cdots ,x_{T}\right)$ and hidden vector sequences $h=\left(h_{1},h_{2},\cdots ,h_{T}\right)$, iterating Equations (4) and 5 from 1 to *T* [37]:

(4) $$h_{t}=H\left(W_{xh}x_{t}+W_{hh}h_{t-1}+b_{h}\right)$$

(5) $$y_{t}=W_{hy}h_{t}+b_{y}$$

where $W_{ij}$ is the weight matrix between layer *i* and *j*, $b_{k}$ is the bias vector for layer *k* and H is the activation function for hidden nodes, usually a sigmoid function $f:R\to R,f\left(t\right)=\frac{1}{1+e^{-t}}$ or a hyperbolic tangent function.

Each cell in the hidden layers of an LSTM has some extra gates to store values, in comparison to other RNN networks: an input gate, forget gate, output gate and cell activation. With the proper combination of these gates, the values propagated through the network can be stored in the long or short term, and easily released to units of the same layer or next layers of the network to improve the capacity of the network with information of past states. The gates of a typical LSTM network are implemented following the equations:

(6) $$i_{t}=\sigma \left(W_{xi}x_{t}+W_{hi}h_{t-1}+W_{ci}c_{t-1}+b_{i}\right)$$

(7) $$f_{t}=\sigma \left(W_{xf}x_{t}+W_{hf}h_{t-1}+W_{cf}c_{t-1}+b_{f}\right)$$

(8) $$c_{t}=f_{t}c_{t-1}+i_{t}\tanh\left(W_{xc}x_{t}+W_{hc}h_{t-1}+b_{c}\right)$$

(9) $$o_{t}=\sigma \left(W_{xo}x_{t}+W_{ho}h_{t-1}+W_{co}c_{t}+b_{o}\right)$$

(10) $$h_{t}=o_{t}\tanh\left(c_{t}\right)$$

where σ is the sigmoid function, *i* is the input gate activation function, *f* the forget gate activation function, *o* is the output gate activation function, and *c* the cell activation function. $W_{mn}$ are the weight matrices from each cell to gate vector. *h* is the output of the unit.

The combination of gates allows an LSTM neural network to decide when to keep or override information in the memory cell, when to access memory cell and when to prevent other units from being perturbed by the memory cell value [39]. More details on the training procedures and the applications of LSTM networks can be found in [44]. The storage of the previous states in the memory of the LSTM can benefit the improvement of the spectrum of speech signals due to their time-dependent nature of past states, which otherwise could not be stored for use in other types of non-recurring neural networks.

## 3. Materials and Methods

The literature related to statistical parametric speech synthesis produced using HMM, have repeatedly reported notable differences with the artificial and the original voices (e.g., [45,46,47]). As described in previous sections, it is possible to reduce the gap between natural and artificial voices by learning a function that maps them directly from the data [29]. In our proposal, we use aligned utterances from natural and synthetic voices produced by the HTS system [48] to establish a correspondence between each frame. Then, LSTM post-filters are trained to map MFCC parameters of synthetic speech into those corresponding to natural speech. For this purpose, our interest is to show the advantage of initializing the weights of the networks in terms of the quality of the results. We initialize the network to establish this advantage, in the following ways:

- Randomly: All the weights have random numbers at the first epoch of training. This initialization method is the most common usage DNN-based post-filtering of artificial speech, and we take it as the base system.
- Initialized-1: The weights are initialized using an auto-associative network (ANN). ANN is a neural network whose input and target vectors are the same [49]. From this definition, it is important to explore whether the ANN can be set from clean and artificial speech spectrum data. Initialized-1 refers to the case of clean speech.
- Initialized-2: The weights of the network are initialized in the form of an ANN trained with artificial speech spectrum vectors.

The ANN was trained with the following procedure: An LSTM network with the same architecture of the post-filters (39 units as inputs and 39 as outputs for the enhancing of MFCC) was trained with the same data at the input and the output in each frame. This way, the network learns the identity function between its inputs and its outputs. After training, the weights of the ANN became the initialized weights of the corresponding post-filters. The indication “1” means the usage of clean data during the pre-training of the LSTM network.

In every case after the pre-training process, the inputs to the LSTM network correspond to the 39 MFCC parameters of each frame for the sentences spoken using the HMM-based voice, while the output corresponds to the MFCC parameters given by the natural voice for the same sentence. Hence, each LSTM post-filter network attempts to solve the regression problem of transforming the values of speech produced by the HMM-based system into those of the natural voices. The experiments allow comparing the two types of initialization: the traditional random approach and the auto-associative proposal (in two cases, using natural or artificial coefficients for the training of the ANN), where the post-filter needs to refine the weights after the described process.

### 3.1. Corpus Description

For the experimentation, we used the CMU Arctic databases, constructed at the Language Technologies Institute at Carnegie Mellon University [50]. They are phonetically balanced and contain the speech of five US English speakers. The databases were designed for research in unit selection speech synthesis, commonly applied also to HMM-based speech synthesis, and consist of around 1150 utterances selected from out-of-copyright texts from Project Gutenberg.

The databases include male and female speakers. A detailed report on the structure and content of the database and the recording conditions is available in the Language Technologies Institute Tech Report CMU-LTI-03-177 18. The five voices that were chosen here for the experiments are identified by a three letters: BDL (male), CLB (female), JMK (male), RMS (male) and SLT (female).

### 3.2. Feature Extraction

The audio files of the database were downsampled to 16 kHz, in order to extract the parameters using the Ahocoder system. In this system, the fundamental frequency $f_{0}^{k}$ (zero-valued if invoiced), 39 MFCC, plus an energy coefficient are extracted from each frame. Hence each frame is represented by a 41st dimensional vector $V_{k}=\left[f_{0}^{k},e^{k},mfcc_{k}^{1},\cdots ,mfcc_{k}^{39}\right]$. For this work, we only kept the 39 MFCC of the parametrization, whilst the remaining parameters were leaving unchanged from the HMM-based voice. Further, an audio waveform can be synthesized from a similar set of parameters using the system (Ahodecoder). Details on the parameter extraction and waveform regeneration of this system can be found in [51].

### 3.3. Experiments

Each of the five voices was parameterized, and the resulting set of vectors was divided into training, validation, and testing sets. The amount of data available for each voice is shown in Table 1. Despite all voices uttering the same phrases, the length differences are due to variations in the speaker’s rate. The test set consists of 50 utterances. The stop criteria of the training process were established in terms of a maximum number of epochs (500) or 25 epochs since the last lower sse value for the validation set.

The AAN initialization was performed with the parameters of all the voices, i.e., there are 10 different cases:

- Five cases where the set of ANN was trained using clean MFCC coefficients (one for each voice). In the results, these cases are identified with the superscript 1.
- Five cases where the set of ANN was trained using MFCC coefficients from the HMM-based voices (one for each voice). In the results, these cases are identified with the superscript 2.

Additionally, to establish a comparison to the traditional random initialization, we performed the post-filtering with three LSTM networks trained independently with random weight initialization.

The LSTM post-filter architecture was defined after a process of trial and error, using the Currennt system [52]. The final selection of three hidden layers, with 150, 100 and 150 units in each one respectively was considered after producing the best results in the first set of experiments, and also having manageable training time for the 25 LSTM networks used in the present work (10 for the initialization of both cases of ANN and 15 for the three-time random initialization).

The training procedure was accelerated by an NVIDIA GPU system and took a mean time of 12 h to train each LSTM. For the whole set of experiments, there was a running time of more than 12 days.

In order to determine the improvement in the efficiency of the supervised pre-training, we use the following objective measures:

- Number of epochs: The time taken to train a neural network is directly associated with the amount of epochs in training. Each epoch consists in a forward and backward pass of data, and the updating of the weights. The lesser epochs it takes to train a network, the less time is needed, so the procedure is more effective.
- sse: This is a common measure for the error in the validation and test sets during training. Is defined as:
  (11) $$\begin{array}{ccc} sse(\theta ) & = & \sum_{n=1}^{T}{\left(c_{x}-\hat{c_{x}}\right)}^{2} \end{array}$$
  (12) $$\begin{array}{cc} = & \sum_{n=1}^{T}{\left(c_{x}-f\left(c_{x}\right)\right)}^{2} \end{array}$$
  where $c_{x}$ is, ${\hat{c}}_{x}$ is *T* the number of frames, and *f* the function the networks perform between its inputs and its outputs. A lower value of sse means that the network is producing outputs more closely related to the objective parameters (those of the natural voice).

Additionally, to verify whether the results represent improvement in the quality of the speech, we incorporate the following measure:

- PESQ: This measure uses a psychoacoustic model to predict the subjective quality of speech. This measure is defined in the ITU-T recommendation P.862.ITU. Results are given in interval [0.5,4.5], where 4.5 corresponds to a perfect reconstruction of the signal.
  PESQ is computed as [53]:
  (13) $$PESQ=a_{0}+a_{1}D_{ind}+a_{2}A_{ind}$$
  where the $D_{ind}$ is the average disturbance and $A_{ind}$ the asymmetrical disturbance. The $a_{k}$ are chosen to optimize PESQ in measuring speech distortion, noise distortion and overall quality.

The results and analysis are shown in the following sections.

## 4. Results

The results are divided into two sections: The first part emphasizes the improvement achieved in the efficiency of the LSTM networks, while the second part shows the results of the metrics used to determine the improvement in the quality of the voices due to the post-filter process. In all cases, the ANN training was stopped by the criteria of a maximum number of epochs (500).

### 4.1. Training Efficiency

In terms of the efficiency, the lower number of epochs is associated with less time in the training procedure, while the lower value of sse means a better performance of the network for the task of regression from the synthetic parameters to the natural ones.

Table 2 presents the results of both the number of epochs and sse for the five voices. The values reported in this table correspond to the best case of the three random initialization and the initialization of the network with natural data (ANN-1). In the SLT voice, the MFCC post-filter required 95 fewer epochs to train with the auto-associative initialization (29% less time), with a benefit of lowering the sse from 290.00 to 276.33.

These results show a more efficient and effective way of training with the supervised initialization. The rest of the voices present improvements in the training time in 43% (BDL voice), 32% (CLB voice), 25% (RMS voice) and 14% (JMK voice). The sse values present improvements in all the cases where the ANN initialization was applied.

**Figure 1:**
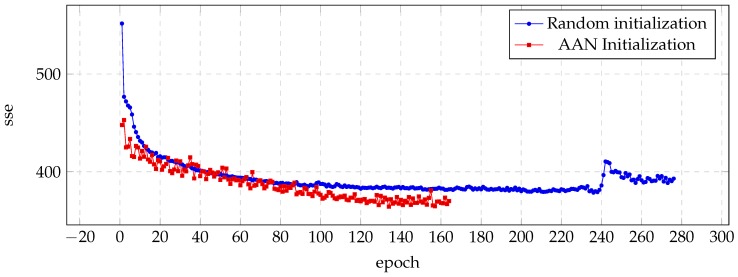
Evolution of the sse value for the validation set during the training process of the BDL voice.

Figure 1 shows the evolution of sse in each training epoch, and illustrates how the auto-associative initialization reaches lower sse with fewer epochs. It can be noticed also how the first epochs represent the greater differences during the training process.

**Figure 2:**
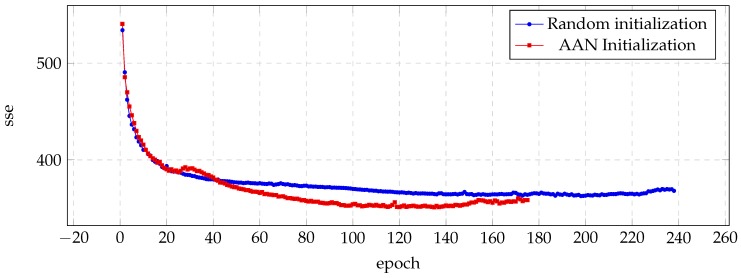
Evolution of the sse value for the validation set during the training process of the CLB voice.

The results of the sse measure for the CLB voice are shown in Figure 2. This figure illustrates how the auto-associative initialization reaches lower sse values with fewer epochs. During the first part of the process (about 40 epochs), the benefits are not noticeable yet, but at the end of the training, they are quite remarkable.

**Figure 3:**
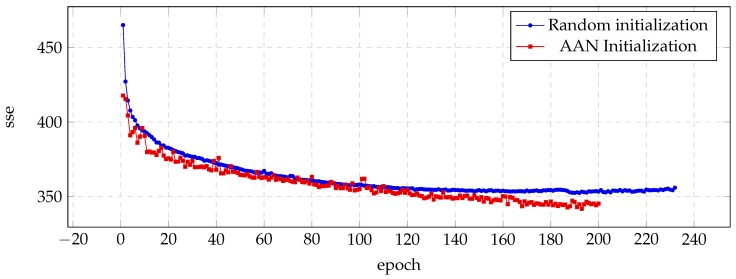
Evolution of the sse value for the validation set during the training process of the JMK voice.

**Figure 4:**
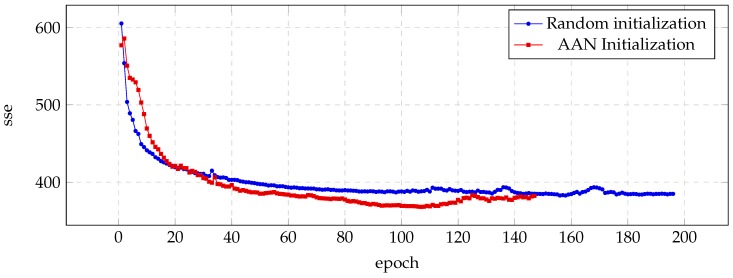
Evolution of the sse value for the validation set during the training process of the RMS voice.

Similar results are presented in Figure 3 (JMK voice) and Figure 4 (RMS voice), with most significant improvements in the case of the JMK voice.

**Figure 5:**
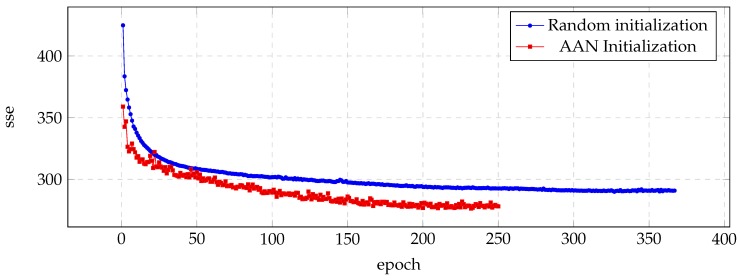
Evolution of the sse value for the validation set during the training process of the SLT voice.

Finally, one of the most significant results is shown in Figure 5, where the evolution of the sse value for the validation set is noticeable during the complete process.

### 4.2. Quality of the Results

Apart from the improvements in the efficiency in terms of objective measures of training time and sse, the convenience of applying the initialization of the LSTM post-filters with the ANN should be tested in terms of the quality of the post-filtering process, which pretends to improve the spectrum of synthetic voices.

Since initialization requires a previous step of training, it is important to establish the independence of the results of the data used in the process. For this reason, in this part of the results, the PESQ was applied to the 50 utterances of the test set considering the case where initialization was performed using natural or synthetic speech parameters. In addition, it is important to compare the results to the random initialization that is considered the base case.

Table 3 presents the result for the case of BDL voice. Here, the best mean value of the PESQ was obtained with the network pre-trained with synthetic parameters of the RMS voice.

Table 4 shows the result for the case of CLB voice. Here, the best mean value of the PESQ was obtained with the network pre-trained with synthetic parameters of the RMS voice. The best results were achieved with the initialization of the post-filter using natural parameters of the RMS and SLT voices and synthetic parameters of the RMS voice.

The PESQ results of the JMK voice are presented in Table 5. The best result was obtained with the ANN initialization performed with natural parameters of the BDL voice, and the second to best with artificial parameters of the JMK voice.

The only case where the ANN initialization of the LSTM post-filter did not achieve a better result than the random initialization, was the RMS voice. These results are shown in Table 6.

Finally, the results for the SLT voice are presented in Table 7. The best results were obtained with the ANN initialization performed with data from the JMK voice.

**Figure 6:**
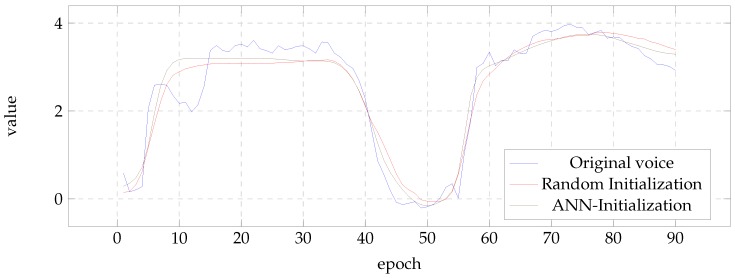
First MFCC for the BDL voice.

**Figure 7:**
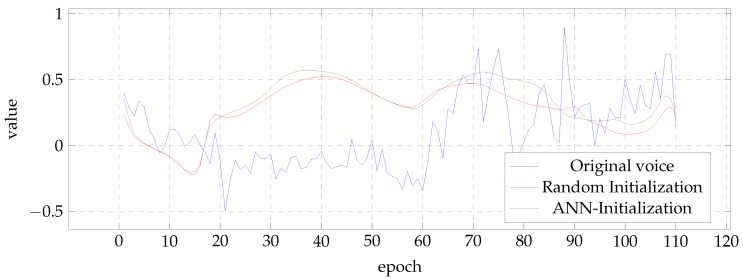
Sample contour of the fifth MFCC for the SLT voice.

To illustrate the effect of the initialization in the results, Figure 6 and Figure 7 show the evolution of the first MFCC coefficient of the BDL and the fifth of the SLT voice, respectively. In all cases, the figures present the result of the best case according to PESQ measure. It can be seen that the results of the post-filters initialized with ANN tend to present values closer to those of the natural voice.

## 5. Discussion

The advantage of proposed ANN-initialization lies on fewer training epochs required to achieve even better sse values than the usual random initialization. All the cases analyzed allow to confirm this benefit of the proposal.

As shown in Table 2, the LSTM post-filters presented significant variations in the number of epochs required for training. These differences can be partially explained by the fact that the quality of synthetic speech relies on the characteristics of the database. This fact means it is expected in the results noticeable differences between the natural and synthetic voices, which can be represented as a bigger or lower gap to map between them in the post-filters.

According to the results in terms of the PESQ measure, neither the number of epochs nor the sse values can be used as a substitute for quality measures of the results. Table 6 is the main evidence among the experiments, which reflects that even though the ANN initialization lowered sse values with fewer epochs, it failed in presenting a better PESQ result than the random initialization. Given that quality measurement is being carried out on the complete speech wave, it is important to emphasize that only the MFCC coefficients are being processed, while the energy, aperiodic coefficients, and $f_{0}$ remained the same, and also have a significant effect on the measurements quality.

Regarding the independence of the data utilized in the pre-training, the results of the BDL voice (Table 3) show significant differences between them. For both the initialization with natural and synthetic data, the ANN initialization shows the best and worst results compared to the random initialization. The CLB voice (Table 4) presents the case where the initialization produces better results than the random initialization in most cases. In particular, it is important to remark that the initialization with parameters of the same CLB voice produces the best results of the three random initializations.

Similar results for the two types of initialization are shown for the JMK voice in Table 5. Here, most of the values are better than the worst case of random initialization, and in all cases, the results were better than the worst case of random initialization. In particular, the initialization with data of the same JMK voice results in better PESQ values than the random base case.

For the case of RMS voice, only two cases of initialization produce values lower than the worst case of random initialization. The case of ANN-1 pre-training with the parameters of the same RMS voice can be considered as an exception among the results because they present the lower value.

The SLT voice shows the benefit of the ANN pre-training proposal of the LSTM post-filters, because all the networks, except for one case, present better results and the worst random initialization, and in most cases are equal to or better than the best random case.

It can be noticed, by comparing all the cases studied in this paper, that only using the initialization with JMK synthetic parameters of the LSTM networks, better results than all the random procedures were obtained in four of the five cases. This fact can support one of the most significant results of this work: that the benefits of the pre-training of LSTM networks for the post-filtering of HMM-based voices depend on the quality of the ANN pre-training.

## 6. Conclusions

In this paper, we have presented a proposal of initialization of LSTM post-filters for the enhancement of statistical parametric artificial voices, based on an auto-associative network. We conducted a comparison using parameters of five voices, both male and female, and two well-known measures for the efficiency of the training neural networks and the most relevant measure for the quality of the speech.

The main assumption for applying the initialization was that the regression performed from the artificial to natural parameters of the voice preserves many characteristics from the input to the output, so the usual random initialization of the network is not the most convenient state to reach the set of weights that best suit the mapping. That is why an auto-associative neural network, which learns the identity function in a supervised way during the pre-training stage, is a better approximation to the desired mapping.

The results show that the auto-associative initialization has benefits in terms of less training epochs and less sum of squared errors for all the voices. The proposed initialization seems independent of the type of data (natural or synthetic) used during the pre-training stage. However, better results tend to be obtained from the initialization with synthetic data.

Due to the quality of synthetic voices relying on parameters other than the MFCC, future work should include extending this proposal of initialization of LSTM networks for the rest of the parameters of synthetic speech.

## Figures and Tables

**Table 1:** Amount of data (vectors) available for each voice in the databases.

Voice	Gender/Accent	Total	Train	Validation	Test
BDL	(M) US-English	676,554	473,588	135,311	67,655
SLT	(F) US-English	677,970	474,579	135,594	67,797
CLB	(F) US-English	769,161	538,413	153,832	76,916
RMS	(M) US-English	793,067	555,147	158,613	79,307
JMK	(M) US-English	635,503	541,856	62,135	31,512

(a) JMK voice in US-English was produced from a Canadian English speaker.

**Table 2:** Comparison of the results for the test set during training the Long Short-term Memory (LSTM) networks to enhance MFCC.

**Random initialization**									
SLT		BDL		CLB		RMS		JMK	
Epochs	sse	Epochs	sse	Epochs	sse	Epochs	sse	Epochs	sse
327	290.00	236	378.58	198	362.56	196	382.80	232	352.36
**ANN initilization 1**									
SLT		BDL		CLB		RMS		JMK	
Epochs	sse	Epochs	sse	Epochs	sse	Epochs	sse	Epochs	sse
232	276.33	134	364.28	135	350.92	147	368.07	200	341.72

**Table 3:** Mean PESQ Results for BDL voice. Higher values represent better results. * is the best result. The superscript 1 means that the LSTM post-filter was pre-trained as ANN using natural parameters, while the superscript 2 means the same procedure applied with synthetic parameters.

Random-Worst	Random-Best	BDL ^1^	CLB ^1^	JMK ^1^	RMS ^1^	SLT ^1^
1.45	1.46	1.45	1.46	1.45	1.44	1.46
		**BDL ^2^**	**CLB ^2^**	**JMK ^2^**	**RMS ^2^**	**SLT ^2^**
		1.43	1.45	1.47	1.49 *	1.47

**Table 4:** Mean PESQ Results for CLB voice. Higher values represent better results. * is the best result. The superscript 1 means that the LSTM post-filter was pre-trained as ANN using natural parameters, while the superscript 2 means the same procedure applied with synthetic parameters.

Random-Worst	Random-Best	BDL ^1^	CLB ^1^	JMK ^1^	RMS ^1^	SLT ^1^
1.16	1.19	1.18	1.20	1.20	1.23*	1.23 *
		**BDL ^2^**	**CLB ^2^**	**JMK ^2^**	**RMS ^2^**	**SLT ^2^**
		1.20	1.20	1.22	1.23 *	1.18

**Table 5:** Mean PESQ Results for JMK voice. Higher values represent better results.

Random-Worst	Random-Best	BDL ^1^	CLB ^1^	JMK ^1^	RMS ^1^	SLT ^1^
1.51	1.56	1.59 *	1.53	1.56	1.56	1.54
		**BDL ^2^**	**CLB ^2^**	**JMK ^2^**	**RMS ^2^**	**SLT ^2^**
		1.55	1.55	1.58	1.55	1.55

**Table 6:** Mean PESQ results for RMS voice. Higher values represent better results.

Random-Worst	Random-Best	BDL ^1^	CLB ^1^	JMK ^1^	RMS ^1^	SLT ^1^
1.70	1.72 *	1.71	1.71	1.71	1.68	1.71
		**BDL ^2^**	**CLB ^2^**	**JMK ^2^**	**RMS ^2^**	**SLT ^2^**
		1.71	1.69	1.70	1.70	1.70

**Table 7:** Mean PESQ Results for SLT voice. Higher values represent better results.

Random-Worst	Random-Best	BDL ^1^	CLB ^1^	JMK ^1^	RMS ^1^	SLT ^1^
0.95	0.97	0.95	0.97	0.98 *	0.95	0.96
		**BDL ^2^**	**CLB ^2^**	**JMK ^2^**	**RMS ^2^**	**SLT ^2^**
		0.97	0.97	0.98 *	0.97	0.94

## Abbreviations

The following abbreviations are used in this manuscript:

AAN	Auto-associative Network
DNN	linear dichroism
HMM	Three letter acronym
LSTM	Multidisciplinary Digital Publishing Institute
PESQ	Directory of open access journals
RBM	Restricted Boltzman Machine
RNN	Recurrent Neural Network
TTS	Text-to-Speech Synthesis
$f_{0}$	Fundamental frequency
